# Biomedical and Forensic Applications of Combined Catalytic Hydrogenation-Stable Isotope Ratio Analysis

**Published:** 2007-09-06

**Authors:** Mark A. Sephton, Will Meredith, Cheng-Gong Sun, Colin E. Snape

**Affiliations:** 1 Department of Earth Science and Engineering, South Kensington Campus, Imperial College London, SW7 2AZ.; 2 School of Chemical, Environmental and Mining Engineering, University of Nottingham, University Park, Nottingham, NG7 2RD, U.K.

**Keywords:** fatty acids, steroids, stable isotopes, hydropyrolysis

## Abstract

Studies of biological molecules such as fatty acids and the steroid hormones have the potential to benefit enormously from stable carbon isotope ratio measurements of individual molecules. In their natural form, however, the body’s molecules interact too readily with laboratory equipment designed to separate them for accurate measurements to be made. Some methods overcome this problem by adding carbon to the target molecule, but this can irreversibly overprint the carbon source ‘signal’. Hydropyrolysis is a newly-applied catalytic technique that delicately strips molecules of their functional groups but retains their carbon skeletons and stereochemistries intact, allowing precise determination of the carbon source. By solving analytical problems, the new technique is increasing the ability of scientists to pinpoint molecular indicators of disease, elucidate metabolic pathways and recognise administered substances in forensic investigations.

## Introduction

Stable isotope ratio measurements are becoming important for methods aimed at determining the origin of organic molecules found in biological fluids. In particular the relative abundance of stable isotopes of carbon (carbon-12 and carbon-13) is very useful and can be determined by modern instruments at high sensitivities and high precision.

During analysis materials are usually converted by combustion to CO_2_ and the ratios of ^13^C to ^12^C in the gas are determined by mass spectrometry (combustion-MS). Variations in values are quoted differentially (compared to an internationally agreed standard, PDB) where: δ^13^C = [(^13^C/^12^C) sample/(^13^C/^12^C) standard −1] × 1000 in per mil (parts per thousand; symbol ‰).

In recent times, methods have been developed where stable isotope ratios can be determined for individual molecules, a procedure termed compound specific isotope analysis (abbreviated to CSIA). This is accomplished using a relatively new development of the carbon isotope ratio technique that combines a separation procedure (gas chromatography, GC) with mass spectrometry (MS) via a combustion interface (Matthews and Hayes, 1978). The integrated methodology, termed GC-C-IRMS, can provide high precision (±0.2‰) measurements on small samples (nanogram quantities) of individual compounds separated from mixtures.

Yet despite the continued development of mass spectrometers that achieve new and unprecedented levels of sensitivity and precision for individual molecules, sample preparation methods for biological molecules represent a bottleneck in analytical advancement. A recent reviewer stated “despite their importance for high-precision compound specific isotope analysis, dedicated studies addressing the issues of sample preparation are few and far between” (Meier Augenstein, 1999).

**Figure 1.**
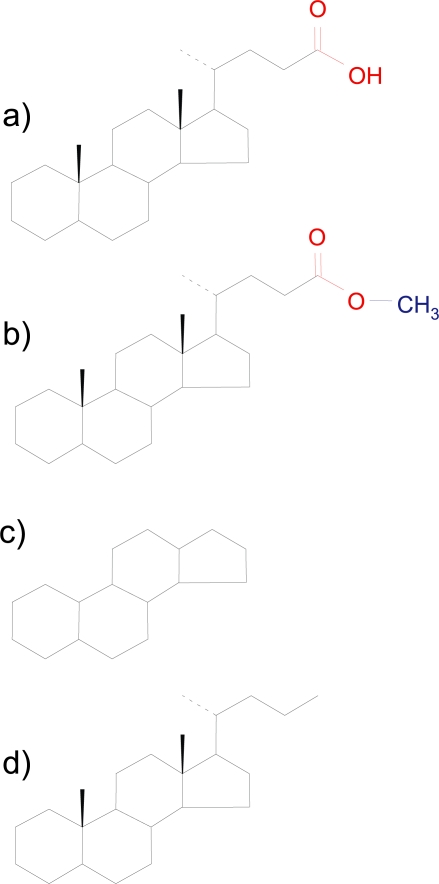
Strategies to make biological molecules such as fatty acids and steroids amenable to stable isotope ratio analysis by GC-C-IRMS. **a)** A common steroid molecule (cholanoic acid) with a functional group (in red) that causes it to interact too readily with the separation equipment. A successful isotope ratio analysis must measure the carbon isotope ratio of the carbon skeleton (in black). **b)** One approach is to modify the functional groups by attaching small molecules (blue) but this adds carbon. **c)** Another approach is to remove functional groups by catalytic reactions but this has previously removed parts of the carbon skeleton. **d)** The hydropyrolysis approach removes functional groups but retains the carbon skeleton intact for stable isotope analysis.

The problem arises because, in their natural form (Fig. 1a) biological molecules interact too readily with the laboratory equipment designed to separate them prior to isotope ratio analysis. Existing strategies to avoid separation problems involve attaching small molecules to the functional groups (Meier Augenstein, 1999) that reduce the ability of functional groups to interact with the separation equipment (Fig. 1b). This derivatization approach solves the separation problem but the addition of extra carbon atoms can corrupt the original carbon isotope signal of the target molecules. Stable isotopic changes occur when derivatization takes place in a non-quantitive manner. Commonly applied derivatization methods include esterification, acetlyation and silylation (Meier Augenstein, 2004). Esterification and acetlyation involve modification of carbon atoms and can induce kinetic isotope effects. Silylation can produce derivatives that interfere with the conversion of the target molecule to CO_2_, a necessary step for CSIA by GC-C-IRMS. Other potentially useful methods remove the functional groups of biological molecules by chemical treatments such as catalytic hydrogenation to convert starting compounds to the parent hydrocarbon and/or the next lower homolog (Fig. 1c). Regrettably, in the past, this approach has removed part of the carbon skeleton of the molecule and information about the structure of the molecule is lost making the products difficult to identify (Beroza, 1962). Furthermore, following the loss of carbon, the carbon isotope ratios of the products may become dissimilar to that of the starting material.

**Figure 2.**
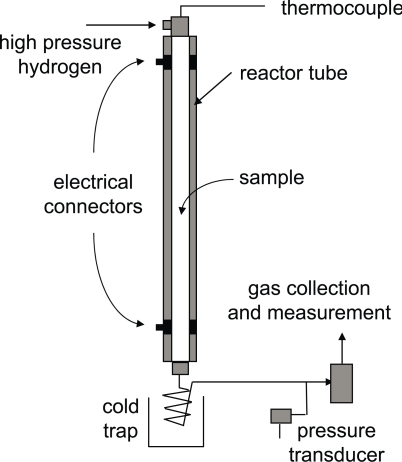
Schematic of the hydropyrolysis equipment.

A new analytical method has been developed to replace the functional groups of biological molecules with hydrogen but retain the carbon skeleton and stereochemistry of the molecule intact (Fig. 1d). Hydropyrolysis involves the catalytic addition of hydrogen to the carbon skeleton at relatively high pressures using a dispersed molybdenum catalyst. Indeed, during hydropyrolysis, all that is lost from the molecule is that which analysts would want to remove prior to carbon isotopic analysis. During the procedure a catalyst, ammonium dioxydithiomolybdate [(NH_4_)_2_MoO_2_S_2_], decomposes in situ above 250 °C to form a catalytically-active molybdenum sulphide phase. Hydropyrolysis experiments are performed in a continuous flow temperature-programmed reactor configuration (Fig. 2), which has been described in the literature (Love et al. 1995, 1997; Russell et al. 2004; Sephton et al. 2005a), with products rapidly swept from the reactor to a silica trap.

Two of the most important classes of molecule starting to benefit from stable isotope studies are the fatty acids and steroid hormones. In their natural form both types of molecule contain functional groups that hinder chromatographic separation during GC-C-IRMS. This paper summarizes and extends previously published work on these molecules (Sephton et al. 2005a; Sephton et al. 2005b).

## Fatty Acids

Fatty acids are one of the most important classes of molecules in metabolism. Fatty acids serve as precursors in the synthesis of other compounds, act as a high density source of calories and facilitate the transport of essential nutrients such as fat-soluble vitamins. Furthermore, some diseases involve disturbances in fatty acid metabolism including diabetes mellitus, sudden infant death syndrome and Reye’s syndrome.

**Figure 3.**
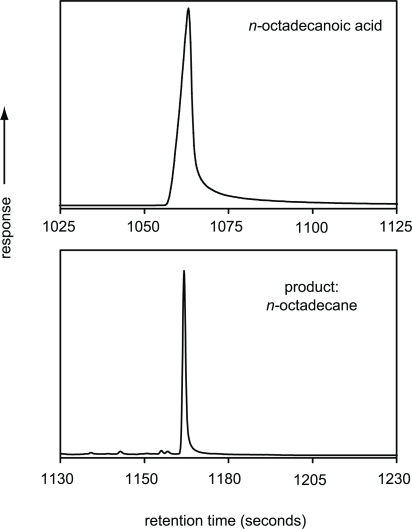
GC-C-IRMS traces of n-octadecanoic acid and its hydropyrolysis product, n-octadecane, displaying a marked increase in chromatographic performance (Sephton et al. 2005a).

To examine the efficacy of hydropyrolysis for transforming functionalised molecules to their hydrocarbon counterparts Sephton et al. (2005a) tested the procedure on a simple fatty acid (*n-*octadecanoic or “stearic” acid). The isotopic composition of this starting material was analysed by combustion-IRMS (Table 1). The *n-*octadecanoic acid was prepared for GC-C-IRMS by subjecting it to hydropyrolysis at 520 ºC. *n-*Octadecanoic acid was adsorbed onto silica and mixed with quartz sand before the catalyst was added. Samples were then heated resistively from 50 ºC to 250 ºC at 30 ºC min^−1^, and then from 250 ºC to 520 ºC at 8 ºC min^−1^, under a hydrogen pressure of 15 MPa. A hydrogen sweep gas flow of 10 dm^3^ min^−1^, measured at ambient temperature ensured that the products were quickly removed from the reactor vessel. Fig. 3 displays GC-C-IRMS analyses of the untreated *n-*octadecanoic acid and its converted counterpart, *n-*octadecane (Sephton et al. 2005a).

Fig. 3 reveals that *n-*octadecanoic acid displayed significant peak tailing. It follows that when analysed within a complex mixture, this peak tailing would lead to peak overlap precluding accurate stable isotope ratio measurements. Broader peaks also increase the proportion of background measured with the analyte, degrading the ultimate stable isotope ratio determination. In contrast, the hydropyrolysis product (*n-*octadecane) gave a much sharper peak that would be less likely to overlap with those for other compounds when present within biological fluids and would produce a measurement with relatively a low contribution from background. In summary, the benefits of the procedure for fatty acid anayses appear to be (i) more precise measurements and (ii) smaller amounts of sample required, both owing to the increased signal to noise ratio associated with hydropyrolysis products relative to their starting materials.

Comparison of carbon isotopic determinations for the untreated *n-*octadecanoic acid by combustion-IRMS and the products from hydropyrolysis by GC-C-IRMS (Fig. 3, Table 1), indicated that the isotopic composition of the processed sample was representative of the starting material. It appears that no isotopic effects are associated with the conversion from acid to alkane. Thus, the technique allows the effective determination of the carbon isotopic composition of individual fatty acids without the use of derivatizing agents.

## Steroid Hormones

Steroid hormones perform vital biochemical functions including regulation of sexual development and function, suppression of inflammation, stress control, and maintenance of salt and water balance. Understanding the origin and fate of individual steroids within the human is essential for both endocrine studies and forensic investigations.

In biochemical investigations of the endocrine system, “labelled” steroids are deliberately enriched in the heavy stable isotope of carbon and are tracked as they pass along their metabolic pathways (e.g. Wolthers and Kraan, 1999). Stable isotope tracers have significant advantages over more conventional radioactive counterparts because they have no negative physiological effects (Koletzko et al. 1997).

Forensic investigations in athletics use natural abundances of stable carbon isotopes to determine the origin of androgenic and anabolic steroids and their metabolites in biological fluids. For example, androgenic-anabolic steroids are part of the World Anti-Doping Agency’s prohibited list (WADA, 2006). The abuse of these substances can be difficult to detect because steroids such as testosterone are found naturally in the body and exogenous analogues may be used to top up normal levels. Moreover, some steroids have uncertain origins and controversy exists about whether they are produced endogenously. For instance, there is increasing evidence that nandrolone metabolites can appear in the urine of people without the administration of exogenous nandrolone (Kohler and Lambert, 2002). Other steroids may be ingested in contaminated meat products or dietary supplements (Debruyckere et al. 1992). Fortunately for the forensic scientist, steroids destined for pharmaceutical applications are produced by modifying steroid molecules from plants (Coppen, 1979). Plants and humans are isotopically distinct and stable isotope studies can discriminate between exogenous and endogenous sources. GC-C-IRMS has been applied with some success to individual steroids in urine samples from humans (e.g. Becchi et al. 1994).

Hydropyrolysis of steroids represents a significant analytical challenge owing to their structural complexity, both in terms of oxygen functionality and the presence of carbon double bonds. To assess the efficacy of hydropyrolysis for steroids Sephon et al. (2005b) subjected 5β cholanic acid, 5α cholestanol and cholesterol to the hydropyrolysis procedure previously described for fatty acids.

**Figure 4.**
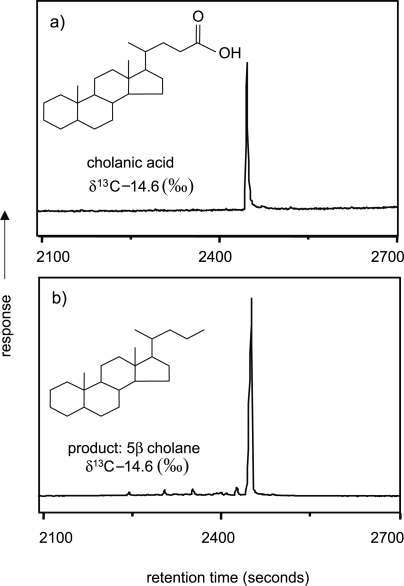
GC-C-IRMS traces of (**a**) cholanic acid and (**b**) its hydropyrolysis product 5b cholane displaying an increase in chromatographic performance.

GC-C-IRMS analyses indicated that hydropyrolysis of 5β cholanic acid produced the single molecular product 5β cholane (Fig. 4). The efficient conversion of 5β cholanic acid to its hydrocarbon counterpart was expected owing to the position of the oxygen functionality on an aliphatic side chain. Hence the conversion is similar to that observed for n-octadecanoic acid.

**Figure 5.**
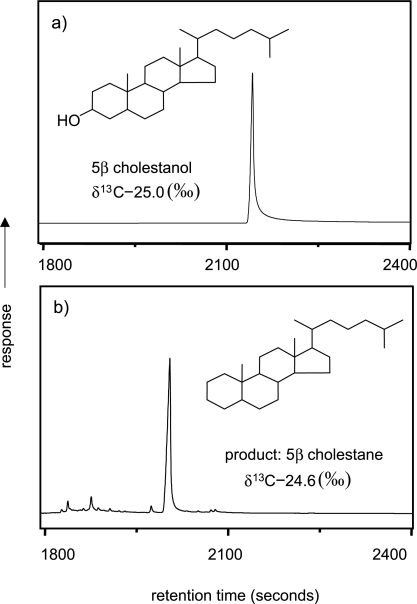
GC-C-IRMS traces of (**a**) 5α cholestanol and (**b**) its hydropyrolysis product 5α cholestane displaying an increase in chromatographic performance (Sephton et al. 2005b).

GC-C-IRMS analysis of the hydropyrolysis products of cholestanol also displayed largely a single molecular product in 5α cholestane (Fig. 5). The results illustrated that, in addition to defunctionalizing aliphatic side chains, hydropyrolysis also efficiently eliminates exocyclic oxygen-containing functional groups.

**Figure 6.**
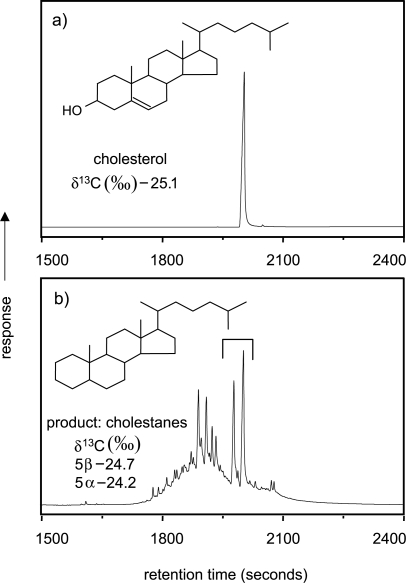
GC-C-IRMS traces of (**a**) cholesterol and (**b**) its hydropyrolysis products (mainly 5b and 5a cholestane) (Sephton et al. 2005b).

For cholesterol, effective hydrogenation would be expected to produce two cholestane isomers (5α and 5β) owing to the non-selective nature of the hydrogenation reaction for the carbon double bond adjacent to ring-joining positions. However GC-C-IRMS analysis of the products (Fig. 6) indicated that extensive rearrangement occurs with the hydropyrolysis procedure giving, in addition to the two expected cholestane isomers, four diasteranes, and an unresolved complex mixture comprising other diasteranes, cholestenes and diasterenes. Multiple products are less suitable for effective carbon isotope analysis and the cholesterol data suggested that a catalyst system and temperature regime that minimises such rearrangements should be a high priority for future development.

Comparison of carbon isotopic determinations for the untreated cholanic acid, cholestanol and cholesterol by combustion-IRMS and for the products from hydropyrolysis by GC-C-IRMS (Table 1), indicated that the isotopic compositions of the processed samples were faithful expressions of the starting materials. It appears that no isotopic effects were associated with the conversion from functionalized steroid to the hydrocarbon counterpart. The technique seems to enable carbon isotopic compositions of individual steroids to be obtained without the corrupting effects associated with derivatization.

## Conversion Results

With the possibility of structural rearrangements occurring during hydropyrolysis (Fig. 6) Sephton et al. (2005b) attempted to constrain the isotopic effects of partial conversion of a functionalised molecule to its hydrocarbon skeleton. This was achieved by operating the procedure at a range of temperatures (400, 500 and 550 °C) with the lower temperatures representing suboptimal conditions. Partial conversion may result in the product exhibiting enrichment in the more reactive ^12^C isotope while the residual starting material becomes enriched in the less reactive ^13^C. Such incomplete transformation could give hydropyrolysis products with carbon isotopic compositions not fully representative of the molecules of interest.

**Figure 7.**
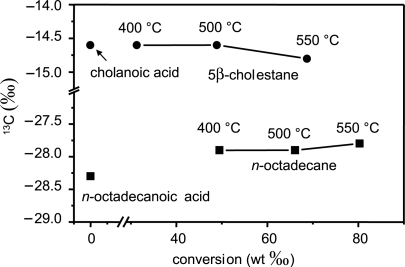
The effect of partial conversion on the carbon isotopic composition of hydropyrolysis products (Sephton et al. 2005b).

To examine the effect of incomplete conversion on stable isotopic compositions, Sephton et al. (2005b) compared the hydropyrolysis products of *n*-octadecane and 5β cholanic acid produced at various temperatures and degrees of conversion. Figure 7 indicates that limited variation occurred between yields of 50 and 80‰ conversion probably reflecting the lack of carbon-carbon bond disruption associated with the procedure. Therefore, even under less than optimal conditions and with only partial conversion, hydropyrolysis products appear to be effective indicators of the carbon isotopic composition of the starting material, suggesting significant analytical contingency in the process.

## Conclusions

Hydropyrolysis shows great potential as an effective preparative technique to facilitate GC-C-IRMS analysis of fatty aids and steroids. The absence of reactions which add or remove carbon prior to analysis make the carbon isotopic compositions of hydropyrolysis products faithful representatives of the starting materials, and the much improved chromatographic performance of the products should allow GC-C-IRMS to be applied to increasingly complex mixtures of fatty acids, steroid hormones and their metabolites. This analytical advance introduces the future possibility of detecting more subtle cases of diseases and endogenous steroid abuse than possible by current methods.

## Figures and Tables

**Table 1.** Carbon isotope ratios of starting materials and hydropyrolysis products. (Data from Sephton et al. 2005a and Sephton et al. 2005b).

**Compound**	δ**^13^C (‰)**	**±1**σ	**Technique**
*Octadecanoic acid*			
*n*-Octadecanoic acid	−28.3	<0.1	Combustion-MS
*n*-Octadecane	−28.0	<0.1	GC-C-IRMS
*Cholanic acid*			
5β-Cholanic acid	−14.6	0.1	Combustion-MS
5β-Cholane	−14.6	0.2	GC-C-IRMS
*Cholestanol*			
5α-Cholestanol	−25.0	0.1	Combustion-MS
5α-Cholestane	−24.6	0.2	GC-C-IRMS
*Cholesterol*			
Cholesterol	−25.1	0.1	Combustion-MS
5β-Cholestane	−24.7	0.3	GC-C-IRMS
5α-Cholestane	−24.2	0.3	GC-C-IRMS
